# Novel Therapeutic Agent against Platelet Activation In Vitro and Arterial Thrombosis In Vivo by Morin Hydrate

**DOI:** 10.3390/ijms19082386

**Published:** 2018-08-13

**Authors:** Chih-Wei Hsia, Ming-Ping Wu, Marappan Velusamy, Chih-Hsuan Hsia, Duen-Suey Chou, Cheng-Lin Tsai, Chia-Yuan Hsu, Thanasekaran Jayakumar, Chi-Li Chung, Joen-Rong Sheu

**Affiliations:** 1 Graduate Institute of Medical Sciences, College of Medicine, Taipei Medical University, Taipei 110, Taiwan; d119106003@tmu.edu.tw (C.-W.H.); mpwu@mail.chimei.org.tw (M.-P.W.); d119102013@tmu.edu.tw (C.-H.H.); gordanmike1003@gmail.com (C.-Y.H.); tjaya_2002@yahoo.co.in (T.J.); 2 Department of Obstetrics and Gynecology, Chi-Mei Medical Center, Tainan 710, Taiwan; 3 Department of Chemistry, North Eastern Hill University, Shillong 793022, India; mvelusamy@gmail.com; 4 Department of Pharmacology, School of Medicine, College of Medicine, Taipei Medical University, Taipei 110, Taiwan; fird@tmu.edu.tw; 5 Graduate Institute of Metabolism and Obesity Sciences, College of Nutrition, Taipei Medical University, Taipei 110, Taiwan; ma48106001@tmu.edu.tw; 6 Division of Pulmonary Medicine, Department of Internal Medical, Taipei Medical University Hospital, Taipei 110, Taiwan

**Keywords:** bleeding time, flavonoid, morin hydrate, OH**·** free radical, platelet activation, protein kinase, thromboembolism

## Abstract

Morin hydrate, a bioactive flavonoid, has been proven to prevent inflammation and apoptosis of cells. Flavonoids can reduce the risk of cardiovascular diseases, in which platelet activation plays a major role. This study investigated the effect of morin hydrate on platelet activation in vitro and in vivo. Morin hydrate markedly inhibited platelet aggregation stimulated by collagen in human platelets but not that stimulated by other agonists. In collagen-activated platelets, morin hydrate inhibited adenosine triphosphate (ATP) release; intracellular Ca^2+^ mobilization; P-selectin expression; and phosphorylation of phospholipase Cγ2 (PLCγ2), protein kinase C (PKC), and Akt. In mitogen-activated protein kinase (MAPK) activation, morin hydrate evidently diminished ERK2 or JNK1 activation, except for p38 MAPK. Additionally, morin hydrate markedly reduced the OH**·** signals in platelet suspensions but not in the cell-free system (Fenton reaction solution). Moreover, morin hydrate substantially increased the occlusion time of thrombotic platelet plug formation but had no effect on bleeding time in mice. In conclusion, morin hydrate crucially inhibits platelet activation through inhibition of the PLCγ2–PKC cascade and subsequent suppression of Akt and MAPK activation, thereby ultimately inhibiting platelet aggregation. Therefore, this paper suggests that morin hydrate constitutes a novel and potential natural therapeutic product for preventing or treating thromboembolic disorders.

## 1. Introduction

**Figure 1:**
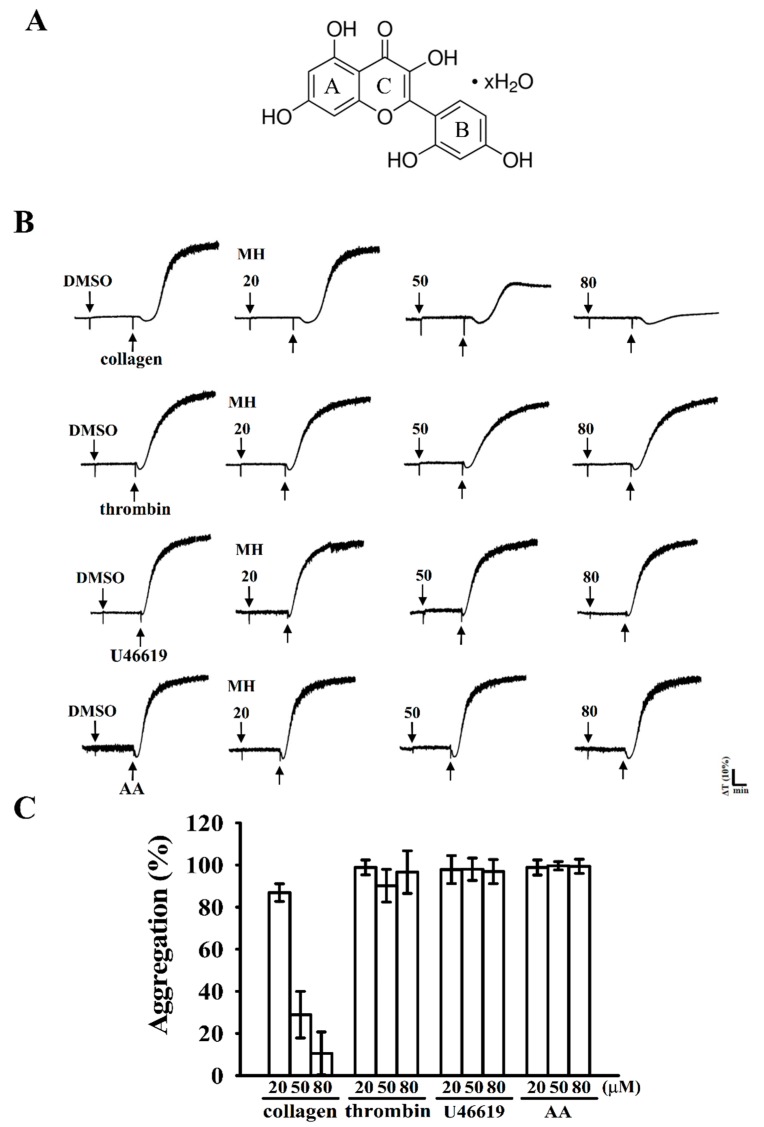
Inhibitory activity of morin hydrate in platelet aggregation stimulated by various agonists in washed human platelets. (**A**) Chemical structure of morin hydrate; (**B**) washed human platelets (3.6 × 10^8^ cells/mL) were preincubated with the solvent control (0.1% dimethyl sulfoxide, DMSO) or morin hydrate (20, 50, and 80 μM) and subsequently treated with 1 μg/mL of collagen, 0.01 U/mL of thrombin, 1 μM of U46619, and 60 μM of arachidonic acid (AA) to stimulate platelet aggregation; (**C**) concentration–response histograms of morin hydrate in inhibition of platelet aggregation (%). All data are presented as means ± standard errors of means (*n* = 4).

Flavonoids are naturally occurring compounds that contain a number of phenolic hydroxyl groups attached to ring structures designated as A, B, and C (Figure 1A). The structure of flavonoids is usually characterized by two aromatic rings (A and B) joined by a three-carbon linked C-pyrone ring (C) to form a C6–C3–C6 skeletal unit (Figure 1A). The bioactivity of flavonoids has attracted academic interest because of its potential health benefits for humans. Diets rich in fruits and vegetables exert protective effects against cardiovascular diseases (CVDs) and certain forms of cancer [1]. Flavonoids have been used as valuable therapeutic agents in modern and traditional medicine.

Morin is a well-known bioactive constituent belonging to the flavonol group and is found in old fustic (*Chlorophora tinctoria*), Osage orange (*Maclura pomifera*), almond, mill (*Prunus dulcis*), fig (*Chlorophora tinctoria*), onion, apple, and other Moraceae, which are used as dietary agents and herbal medicines [2,3]. Morin exhibits various biological activities such as antimutagenesis, anti-inflammation, antiarthritis, and cardioprotective activities, and inhibits xanthine oxidase activity and cell proliferation [4,5,6]. Morin has attracted considerable interest because of its antitumor activity in vitro and in vivo [7].

Dietary factors play key roles in the development and prevention of various human diseases, such as myocardial infarction and ischemic stroke. The risk of CVDs have been reduced with intake of dietary flavonoids [8]. Platelet adherence and aggregation is believed to be initiated intraluminal thrombosis and thus, these events may play a crucial role in atherothrombotic processes in addition to mediating hemostasis [9]. Moreover, platelets are the first-line defense against hemorrhage and important for maintaining the integrity of the vascular system. Upon platelet activation, several mediators such as adenosine triphosphate (ATP) and thromboxane A_2_ are released in conjunction with intracellular Ca^2+^ ([Ca^2+^]i) mobilization. These processes further attract other platelets toward the injured endothelium and therefore cause the thickening of the initial platelet monolayer. Finally, fibrinogen binds to its specific platelet receptor of integrin α_IIb_β_3_, thereby completing the final common pathway for platelet aggregation.

Tzeng et al. [10] demonstrated that morin hydrate exhibited potent bioactivity in the inhibition of rabbit platelet aggregation stimulated by arachidonic acid (AA). In our preliminary study, we observed that 20 µM of morin hydrate significantly inhibited platelet aggregation in washed human platelets. However, the effect of morin hydrate on platelet activation remained to be thoroughly investigated. Therefore, in the current study, we systematically examined in vitro and in vivo effects of morin hydrate on washed human platelets and experimental mice to further characterize the detailed mechanisms of morin hydrate–mediated inhibition of platelet activation.

## 2. Results

### 2.1. Effect of Morin Hydrate on Aggregation of Washed Human Platelets Stimulated by Various Agonists

Morin hydrate (20, 50, and 80 μM; Figure 1B,C) strongly inhibited platelet aggregation stimulated by 1 μg/mL of collagen but not by 0.01 U/mL of thrombin, 1 µM of U46619, a prostaglandin endoperoxide, or AA (60 µM). At 80 μM, morin hydrate almost inhibited platelet aggregation stimulated by collagen. The 50% inhibitory concentration (IC_50_) of morin hydrate for collagen-stimulated aggregation was approximately 40 μM. Therefore, in subsequent experiments, the IC_50_ (40 µM) and maximal concentration (80 µM) of morin hydrate were used to explore the possible mechanisms in human platelets. In addition, the solvent control (0.1% dimethyl sulfoxide, DMSO) did not significantly affect platelet aggregation (Figure 1B).

### 2.2. Regulatory Role of Morin Hydrate in ATP Release, Relative [Ca^2+^]i Mobilization, and Surface P-Selectin Expression in Washed Human Platelets

**Figure 2:**
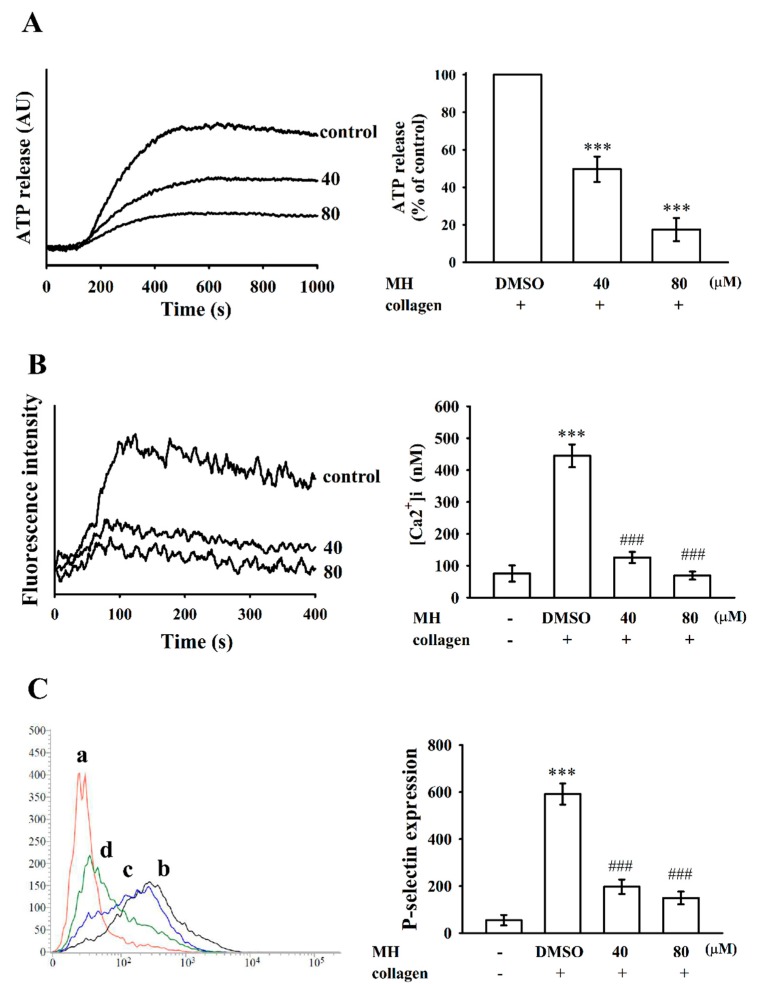
Effects of morin hydrate on adenosine triphosphate (ATP) release, relative intracellular Ca^2+^ ([Ca^2+^]i) mobilization, and surface P-selectin expression in human platelets. Washed platelets (3.6 × 10^8^ cells/mL) were preincubated with the solvent control (0.1% DMSO), morin hydrate (40 and 80 µM), or fluorescein isothiocyanate (FITC)–P-selectin (2 µg/mL); collagen (1 μg/mL) was then added to trigger either: (**A**) ATP release (AU; arbitrary unit), (**B**) relative [Ca^2+^]i mobilization, or (**C**) the fluorescence profiles of (a) FITC only as a resting control; washed platelets were preincubated with the (b) solvent control (0.1% DMSO) or morin hydrate (c, 40 µM; d, 80 µM) for 3 min, followed by the addition of FITC–P-selectin and subsequently treated with collagen to induce platelet activation. The corresponding statistical data are shown on the right panel of each figure (**A**–**C**). Data are presented as means ± standard errors of means (*n* = 4). *** *p* < 0.001 compared with the (**A**) DMSO-treated group or (**B**,**C**) resting control; ### *p* < 0.001 compared with the DMSO-treated group.

Platelet activation is associated with release of granular contents (e.g., ATP and Ca^2+^ from dense granules and P-selectin expression from α-granules). In the present study, morin hydrate (40 and 80 µM) markedly and concentration-dependently hindered ATP release (Figure 2A) and relative [Ca^2+^]i mobilization (resting control, 75.4 ± 25.5 nM; collagen stimulated, 444.9 ± 35.7 nM; 40 μM, 125.9 ± 17.6 nM; 80 μM, 69.1 ± 12.7 nM; *n* = 4; Figure 2B) in platelets stimulated by collagen (1 μg/mL). In resting platelets, P-selectin is located on the inner walls of α-granules. Platelet activation exposes the inner walls of the granules to the outer parts of the cell [11]. Treatment with morin hydrate markedly reduced collagen-induced surface P-selectin expression (resting control, 55.0 ± 22.5; collagen activated, 591.7 ± 44.9; 40 μM, 197.3 ± 30.8; 80 μM, 149.7 ± 27.0; *n* = 4; Figure 2C). The corresponding statistical data are presented on the right panels of Figure 2A–C.

### 2.3. Effects of Morin Hydrate on Cytotoxicity, the PLCγ2–PKC Cascade, and Akt Activation

**Figure 3:**
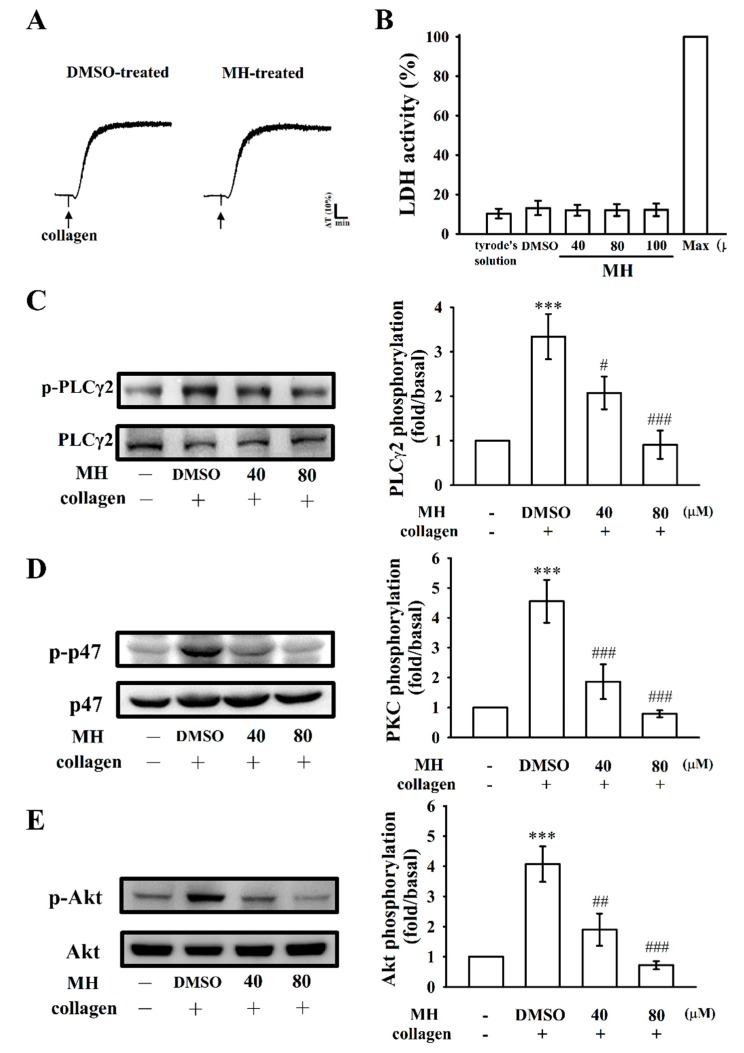
Effects of morin hydrate on cytotoxicity, lactate dehydrogenase (LDH) release, and PLCγ2, PKC, and Akt activation in human platelets. (**A**) Washed platelets (3.6 × 10^8^ cells/mL) were preincubated with the solvent control (0.1% DMSO) or morin hydrate (100 μM) for 10 min and consequently washed twice with Tyrode’s solution; collagen (1 μg/mL) was then added to trigger platelet aggregation; (**B**) washed platelets were preincubated with the solvent control (0.1% DMSO) or morin hydrate (40, 80, and 100 µM) for 20 min and a 10-µL aliquot of the supernatant was deposited on a Fuji Dri-Chem slide LDH-PIII, as described in Materials and Methods. (**C**–**E**) For the other experiments, washed platelets were preincubated with the solvent control (0.1% DMSO) or morin hydrate (40 and 80 µM) and subsequently treated with collagen (1 μg/mL) to induce (**C**) PLCγ2 and (**D**) PKC activation (p47, pleckstrin phosphorylation) and (**E**) Akt phosphorylation. Data are presented as means ± standard errors of means (*n* = 4). *** *p* < 0.001 compared with the resting control; # *p* < 0.05, ## *p* < 0.01, and ### *p* < 0.001 compared with the DMSO-treated group.

The aggregation curves of platelets preincubated with morin hydrate (100 μM) for 10 min and successively washed with Tyrode’s solution were not significantly different from those of platelets preincubated with the solvent control (0.1% DMSO) under equivalent conditions (Figure 3A). This result primarily indicated that the inhibitory effects of morin hydrate on platelet aggregation are reversible and noncytotoxic. In addition, the lactate dehydrogenase (LDH) study revealed that morin hydrate (40, 80, and 100 μM) incubated with platelets for 20 min did not significantly increase the LDH activity in the platelets (Figure 3B); these findings demonstrated that morin hydrate does not affect platelet permeability or induce platelet cytolysis.

Phospholipase C (PLC) hydrolyzes phosphatidylinositol 4,5-bisphosphate to produce the secondary messengers inositol 1,4,5-trisphosphate (IP_3_) and diacylglycerol (DAG). IP_3_ activates relative [Ca^2+^]i mobilization and DAG stimulates protein kinase C (PKC), giving an approximately 47 kDa protein that is principally phosphorylated (p47 protein; pleckstrin) and causes ATP release [12]. As specified, morin hydrate markedly reduced relative [Ca^2+^]i mobilization (Figure 2B). We further examined the effect of morin hydrate on the phosphorylation of the PLCγ2–PKC signaling cascade. As shown in Figure 3C, D, morin hydrate (40 and 80 µM) markedly reduced both PLCγ2 and PKC activation (p47) phosphorylation in collagen-activated platelets. Moreover, Akt is a serine/threonine-specific protein kinase that plays a key role in multiple cellular processes such as cell proliferation, apoptosis, cell migration, and platelet activation [13]. Morin hydrate (40 and 80 µM) evidently inhibited collagen-induced Akt phosphorylation (Figure 3E).

### 2.4. Effect of Morin Hydrate on p38 MAPK, ERK2, and JNK1 Activation

**Figure 4:**
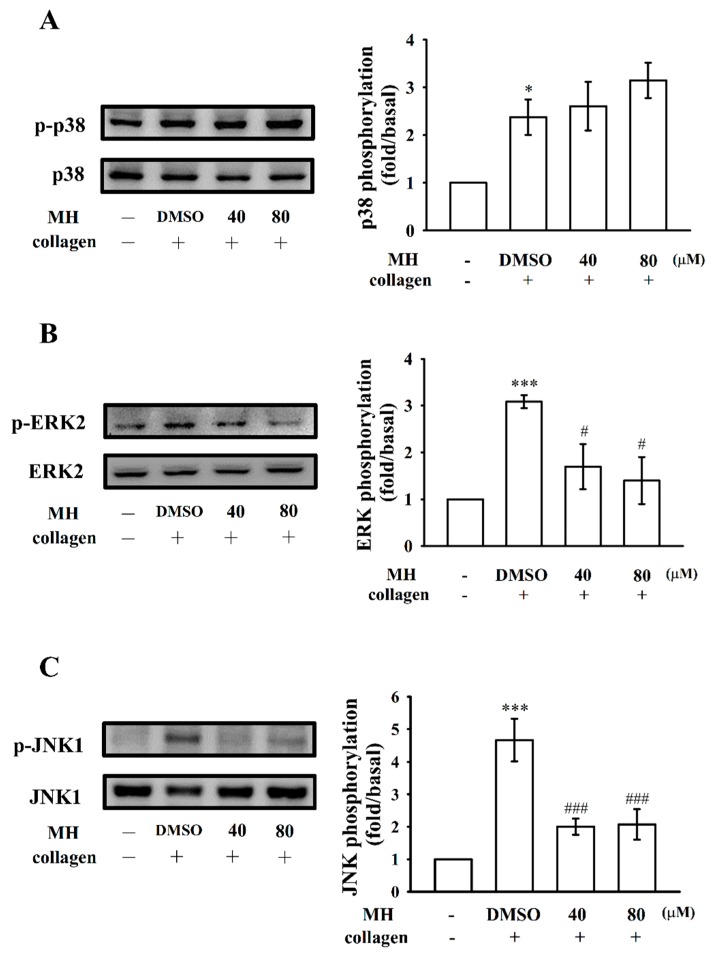
Morin hydrate inhibits p38 MAPK, ERK2, and JNK1 phosphorylation in collagen-activated platelets. Washed platelets were preincubated with the solvent control (0.1% DMSO) or morin hydrate (40 and 80 µM) and subsequently treated with collagen (1 μg/mL) to trigger (**A**) p38 MAPK, (**B**) ERK2, and (**C**) JNK1 activation. Data are presented as means ± standard errors of means (*n* = 4). * *p* < 0.05 and *** *p* < 0.001 compared with the resting control; # *p* < 0.05 and ### *p* < 0.001 compared with the DMSO-treated group.

To further examine the inhibitory mechanisms of morin hydrate, several signaling molecules associated with MAPKs were evaluated. The major kinases, including p38 MAPK, ERKs, and JNKs, regulate cellular responses in eukaryotic organisms and contribute to cell proliferation, migration, differentiation, and apoptosis. ERK2, JNK1, and p38 MAPK have been identified in platelets [14]. As shown in Figure 4A–C, morin hydrate (40 and 80 µM) evidently inhibited ERK2 and JNK1 phosphorylation except for p38 MAPK.

### 2.5. Evaluation of OH·-Scavenging Activity of Morin Hydrate through Electron Spin Resonance (ESR) Spectrometry

**Figure 5:**
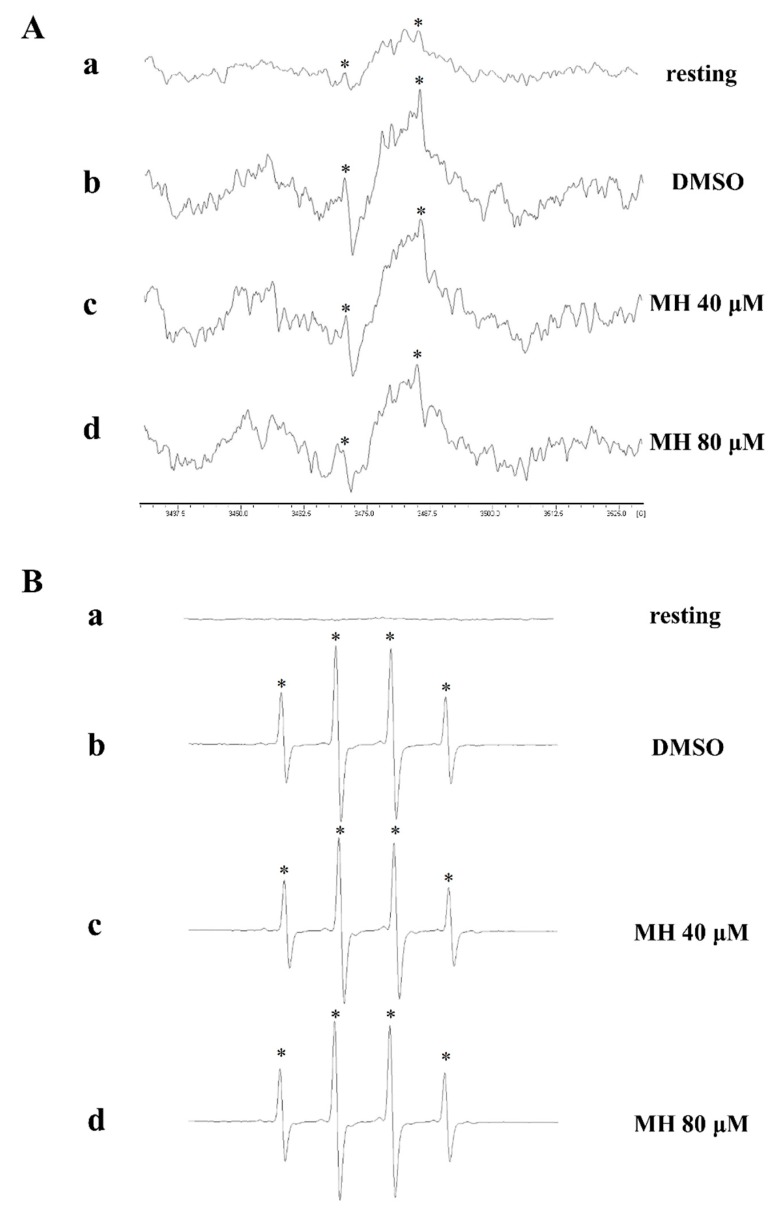
Activities of morin hydrate on OH**·** formation in human platelet suspensions or the Fenton reaction solution. (**A**) Washed platelet suspensions or (**B**) the Fenton reaction solution was preincubated with (a) Tyrode’s solution (resting control), (b) 0.1% DMSO, or morin hydrate at (c) 40 µM or (d) 80 µM. Collagen (1 µg/mL) was then added for the Electron Spin Resonance (ESR) experiments, as described in Materials and Methods. Profiles are representative of four independent experiments, and an asterisk (*) indicates OH**·** formation.

Signals from the ESR study are the indicative of OH**·** radical formation, which was increased in collagen-stimulated platelet suspensions (Figure 5Ab) and the Fenton reaction solution (cell-free system; Figure 5Bb) when compared with the resting control (Figure 5Aa,Ba). Morin hydrate (80 μM) markedly reduced collagen-induced OH**·** signals in the platelet suspensions (Figure 5Ad) but not in the Fenton reaction solution (Figure 5Bd). This result indicated that morin hydrate reduced intracellular OH**·** radical formation but had no significant effect on the cell-free system.

### 2.6. Effects of Morin Hydrate on Platelet Thrombi in Mesenteric Microvessels and Bleeding Time In Vivo

**Figure 6:**
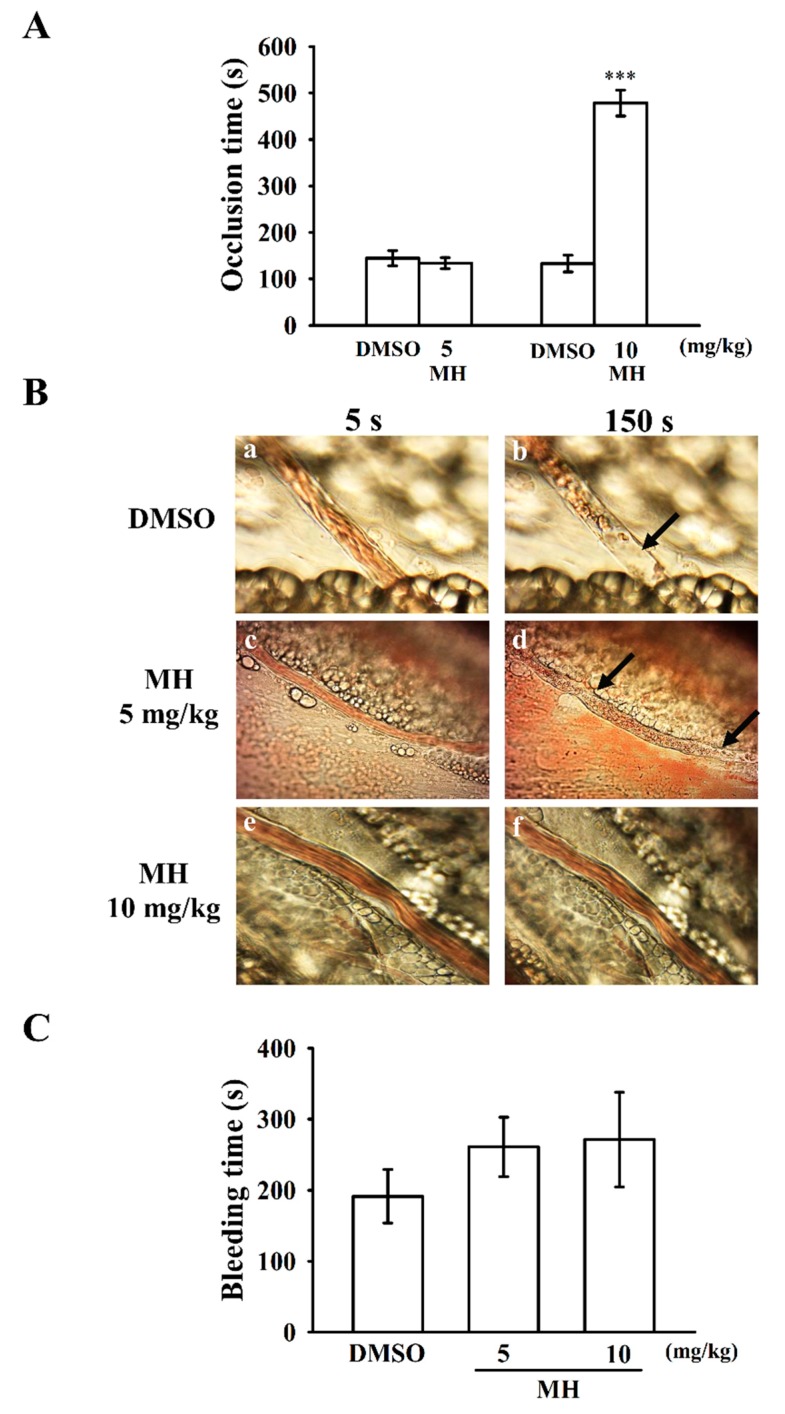
In vivo activities of morin hydrate on thrombotic platelet plug formation in the mesenteric venules and bleeding time in the tail veins of mice. (**A**) Mice were given an intravenous bolus of the solvent control (0.1% DMSO) or morin hydrate (5 and 10 mg/kg) and the mesenteric venules were irradiated to induce microthrombus formation (occlusion time); (**B**) microscopic images (400× magnification) of 0.1% DMSO-treated controls (a and b); the 5 mg/kg (c and d) and 10 mg/kg (e and f) morin hydrate–treated groups were recorded at 5 s (a, c, and e) and 150 s (b, d, and f) after irradiation. The photographs are representative of six similar experiments. The black arrows indicate platelet plug formation; (**C**) bleeding time was measured through transection of the tail in mice after 30 min of administration of 5 or 10 mg/kg of morin hydrate intraperitoneally. Data are presented as means ± standard errors of means (**A**–**C**, *n* = 6). *** *p* < 0.001 compared with the 0.1% DMSO-treated group.

We investigated the effect of morin hydrate on thrombus formation in vivo. The occlusion time in microvessels pretreated with 15 µg/kg of fluorescein sodium was approximately 150 s. When morin hydrate was administered at 10 mg/kg but not 5 mg/kg after pretreatment with fluorescein sodium, the occlusion times were significantly prolonged compared with those of the DMSO-treated controls (DMSO: 144.5 ± 16.1 s vs. 5 mg/kg: 133.9 ± 12.2 s, *n* = 6, *p* > 0.05; DMSO: 133.0 ± 17.9 s vs. 10 mg/kg: 478.4 ± 27.9 s, *n* = 6, *p* < 0.001; Figure 6A). The thrombotic platelet plug was observed in mesenteric microvessels at 150 s (black arrow) but not at 5 s after irradiation in the DMSO-treated group (Figure 6Ba,Bb). When 5 mg/kg morin hydrate was administered, platelet plug formation was also observed at 150 s (black arrow) but not at 5 s after irradiation (Figure 6Bc,Bd). However, upon administration of 10 mg/kg of morin hydrate, the thrombotic platelet plug was not observed at 5 or 150 s (Figure 6Be,Bf). The blood flow rate of the DMSO-treated venule was lower than that of the morin hydrate–treated venule because the platelet plug appeared at 150 s (Figure 6Bb). We also investigated the tail transection model of mice 30 min after administration of 5 or 10 mg/kg morin hydrate intraperitoneally; the bleeding times recorded were 191.3 ± 37.6 s (DMSO-treated group; *n* = 6), 260.7 ± 41.9 s (group treated with morin hydrate, 5 mg/kg; *n* = 6), and 271.3 ± 66.7 s (group treated with morin hydrate, 10 mg/kg; *n* = 6) (Figure 6C). Each mouse was monitored for 15 min after bleeding had stopped to detect any re-bleeding. The results suggested that morin hydrate markedly diminished the platelet plug formation in vivo but had no significant effect on bleeding time.

## 3. Discussion

Our study was the first to demonstrate that morin hydrate shows in vitro antiplatelet effect and effectively inhibit arterial thrombosis in vivo. In general, dietary intake from the natural sources is likely insufficient for attaining plasma concentrations that can inhibit platelet activation in vivo, however, several nontoxic prophylactic agents such as food products and nutritional supplements is ideal for stopping atherothrombotic events. Thus, morin hydrate may signify a novel antithrombotic agent for use in humans.

In the present study, morin hydrate effectively inhibited platelet aggregation stimulated by collagen but not that stimulated by thrombin, AA, or U46619, indicating that this compound inhibits platelet aggregation through a markedly PLC-dependent mechanism. Various physiological stimuli (e.g., thrombin, collagen, ADP, and epinephrine) activate platelets, and these stimulators are believed to exert their effects via interaction with specific receptors on platelet membranes. The matrix protein collagen is present in the vascular subendothelium and vessel wall and acts as a substrate for platelet adhesion; it is also an endogenous platelet activator. Among the platelet receptors known to interact directly with collagen, integrin α_2_β_1_ and glycoprotein (GP) VI [15] appear to play a key role and have recently gained academic attention. GP VI is widely recognized as a requisite factor for formation of platelet aggregates on collagen surfaces under blood flow [16]. Integrin α_2_β_1_ is another major collagen receptor on endothelial cells and platelets. In cells expressing integrin α_2_β_1_, many signals (including tyrosine phosphorylation and matrix remodeling) are activated after cell adhesion to collagen [17]. Recent findings have suggested that integrin α_2_β_1_ and GP VI might contribute to the overall processes of platelet adhesion and activation [18].

Activation of platelets by collagen substantially alters phospholipase activation. PLC activation is an early event in response to numerous extracellular stimuli. Upon activation, PLC produces two crucial second messengers, namely DAG and inositol trisphosphate (IP_3_), both of which play key roles in many signaling pathways, including activation of protein kinase C (PKC) and protein kinase D (PKD) and induction of calcium influx [19]. The signaling axis of PLC/PKC/PKD was shown to play a key role in many signaling pathways [20]. In response to various extracellular stimuli, there are 13 phosphatidylinositide-specific PLCs that are divided into six subgroups: PLCβ, PLCγ, PLCδ, PLCε, PLCξ, and PLCη [21]. The PLCγ family comprises isozymes 1 and 2. PLCγ2 is involved in collagen-dependent signaling on platelets [21]. In our study, morin hydrate diminished the activation of the PLCγ2–PKC cascade stimulated by collagen, suggesting that the morin hydrate–mediated inhibition of platelet activation involves PLCγ2 downstream signaling; moreover, this result explains why morin hydrate was more efficacious in inhibiting collagen induced platelet aggregation than that induced by thrombin, U46619, or AA.

Platelets adhere to the subendothelial matrix protein collagen, which alters their shape and releases granular contents of ATP, Ca^2+^, and P-selectin. P-selectin is an adhesion molecule kept in the platelets α-granules, upon activation it is expressed on a platelet surface membrane and successively expressed on the external membrane through membrane flipping. P-selectin mediates the initial formation of platelet aggregates and simplifies the development of large platelet aggregates [22]. Agonists such as collagen, thrombin, and AA activate [Ca^2+^]i to phosphorylate the Ca^2+^/calmodulin-dependent myosin light chain (20 kDa), which is involved in the secretion of granule contents such as serotonin and ATP [23], as well as platelet aggregation. So, suppression of [Ca^2+^]i mobilization and ATP production is critical for assessing the antiplatelet effects of a compound.

Specific MAPK kinases (MEKs), specifically, MEK1/2, MEK3/6, and MEK4/7 activate MAPKs, such as ERKs, p38 MAPK, and JNKs, respectively [24]. ERK2 activation is involved in platelet aggregation needing prior ATP release, which triggers P_2_X_1_-mediated Ca^2+^ influx and activates ERK2, thereby increasing the phosphorylation of myosin light chain kinase [25]. JNK1 is recently identified in platelets and it activated by several agonists, including collagen, thrombin, and ADP [26], however its role in platelets is poorly recognized. Furthermore, a reduced integrin α_IIb_β_3_ activation and severe granule secretion impairment was proved in JNK^−/−^ platelets [25]. Additionally, cytosolic phospholipase A_2_ (cPLA_2_), a substrate of p38 MAPK activity is induced by agonists such as von Willebrand factor and thrombin [25]. Therefore, p38 MAPK is consider important for cPLA_2_ stimulation [27]. These literatures revealed reasonable explanation is why morin hydrate inhibited ERK2 and JNK1 activation largely than p38 MAPK activation.

Akt is a downstream effector of phosphoinositide 3 (PI3)-kinase. A defective agonist-induced platelet activation was found in Akt-knockout mice, proposing that Akt regulates platelet activation and such regulation may have consequences related to thrombosis [13]. Among the three mammalian Akt isoforms of Akt 1, 2, and 3, the first two isoforms were detected in human platelets [28]. Akt inhibitors have usually reported similar roles of Akt 1 and 2 in human platelet activation. Therefore, protein kinases for Akt activation, particularly PI3-kinase β, may be appropriate targets for the development of antithrombotic therapies. Our previous study found PI3-kinase/Akt and MAPKs are mutually activated as upstream regulators of PKC in activated platelets [29].

Reactive oxygen species (ROS) produced through platelet activation (i.e., H_2_O_2_ and OH**·**) might affect cells that they come into contact with such as endothelial cells, thereby enhancing platelet reactivity during thrombus formation. Free radicals act as secondary signals that increase [Ca^2+^]i levels during the initial phase of platelet activation, and PKC is involved in receptor-mediated production of free radicals in platelets [30]. In addition, H_2_O_2_ produced by platelets is converted into OH**·** because platelet aggregation is inhibited by OH**·** scavengers [30]. Morin was reported to scavenge ROS and free radicals of oxygen in an indirect analysis study [31]. Our ESR spectrometry results provided direct evidence that morin hydrate significantly reduced OH**·** formation in platelet suspensions but had no effect in a cell-free system.

In this study on thrombosis, mesenteric venules were continuously irradiated by fluorescein sodium throughout the experimental period, leading to strong damage to the endothelial cells. Here, 10 mg/kg of morin hydrate significantly prolonged the occlusion times; this effect may be mediated, at least partially, by inhibition of platelet activation. In addition, we used the tail transection mouse model to examine the effects of morin hydrate on bleeding time in vivo. Although aspirin is the most effective antiplatelet drug prescribed for preventing or treating cardiovascular and cerebrovascular diseases, it causes unwanted prolongation of bleeding time. In this model, the bleeding time of the morin hydrate–treated mice was not significantly different to the solvent control, indicating that morin hydrate possesses antiplatelet activity in vivo but had no effect on bleeding time. Liu et al. [32] reported that treatment with 20 mg/kg of morin hydrate markedly reduced myocardial ischemia-reperfusion injury (MIRI) in rats. Although results in animal species and experimental models have differed, morin hydrate evidently possesses a more potent ability to inhibit arterial thrombosis than protect against MIRI.

The findings of the present study revealed that morin hydrate plays a novel role in inhibiting platelet activation in vitro and in vivo, suggesting that it can be used in prophylactic applications. Generally, a nutritional or dietary supplement is required to produce a prophylactic effect in humans. However, selection of doses for time-course treatments may be confounded by variation in responses among users. This study provided new insights into the role of morin hydrate in blocking collagen-specific signaling events involved in platelet activation. However, the involvement of other mechanisms yet to be identified in morin hydrate–mediated inhibition of platelet activation requires investigation.

## 4. Materials and Methods

### 4.1. Chemicals and Reagents

Morin hydrate, thrombin, collagen, AA, luciferin–luciferase, U46619, heparin, prostaglandin E_1_ (PGE_1_), 5,5-dimethyl-1-pyrroline N-oxide (DMPO), and bovine serum albumin (BSA) were purchased from Sigma (St. Louis, MO, USA). Fura-2AM was obtained from Molecular Probes (Eugene, OR, USA). An anti-phospho-p38 mitogen-activated protein kinase (MAPK) Ser^182^ monoclonal antibody (mAb) was purchased from Santa Cruz Biotechnology (Santa Cruz, CA, USA). Anti-p38 MAPK, anti-phospho-c-Jun N-terminal kinase (JNK) (Thr^183^/Tyr^185^), and anti-p44/42 extracellular signal-regulated kinase (ERK) mAbs, as well as anti-phospholipase Cγ2 (PLCγ2), anti-phospho (Tyr^759^) PLCγ2, anti-phospho-(Ser) protein kinase C (PKC) substrate (pleckstrin; p-p47), anti-JNK, and anti-phospho-p44/p42 ERK (Thr^202^/Tyr^204^) polyclonal antibodies (pAbs) were purchased from Cell Signaling (Beverly, MA, USA). Anti-phospho-protein kinase B (Akt) (Ser^473^) and anti-Akt mAbs were purchased from Biovision (Mountain View, CA, USA). An anti-pleckstrin (p47) pAb was purchased from GeneTex (Irvine, CA, USA). A Hybond-P polyvinylidene fluoride (PVDF) membrane, an enhanced chemiluminescence Western blotting detection reagent, horseradish peroxidase (HRP)-conjugated donkey anti-rabbit immunoglobulin G (IgG), and sheep anti-mouse IgG were purchased from Amersham (Buckinghamshire, UK). A fluorescein isothiocyanate (FITC) anti-human CD42P (P-selectin) mAb was obtained from BioLegend (San Diego, CA, USA). Morin hydrate was dissolved in 0.1% dimethyl sulfoxide (DMSO) and stored at 4 °C.

### 4.2. Platelet Aggregation

This study was approved by the Institutional Review Board of Taipei Medical University (N201612050; 20/January/2017) and conformed to the directives of the Declaration of Helsinki. All human volunteers involved in this study provided informed consent. Human platelet suspensions were prepared as described previously [9]. Human blood samples were obtained from adult volunteers who refrained from use of drugs or other substances that could have interfered with the experiment for at least 14 days before sample collection; the collected blood samples were mixed with an acid-citrate-dextrose solution. After centrifugation, platelet-rich plasma (PRP) was supplemented with 0.5 μM of PGE_1_ and 6.4 IU/mL of heparin. Tyrode’s solution containing 3.5 mg/mL of BSA was used to prepare the final suspension of washed human platelets. The final Ca^2+^ concentration in the Tyrode’s solution was 1 mM. A platelet aggregation study was conducted using a lumi-aggregometer (Payton Associates, Scarborough, ON, Canada), as described previously [9]. An isovolumetric solvent control (0.1% DMSO) or morin hydrate was preincubated with platelet suspensions (3.6 × 10^8^ cells/mL) for 3 min before the addition of the agonists (i.e., collagen). The extent of platelet aggregation was calculated as the percentage compared with individual control (without morin hydrate) expressed in light transmission units after the reaction had proceeded for 6 min. For an ATP release assay, 20 μL of luciferin–luciferase was added 1 min before the addition of collagen (1 µg/mL), and the amount of ATP released was compared with that released by the control (without morin hydrate).

### 4.3. Measurement of Relative [Ca^2+^]i Mobilization

The relative [Ca^2+^]i concentration was determined using Fura-2AM, as described previously [9]. Concisely, citrated whole blood was centrifuged at 120× *g* for 10 min and the PRP was collected and incubated with Fura-2AM (5 μM) for 1 h. The Fura-2AM-loaded platelets were preincubated with morin hydrate (40 and 80 µM) in the presence of 1 mM of CaCl_2_ and then stimulated with collagen (1 µg/mL). The Fura-2 fluorescence was measured using a spectrofluorometer (Hitachi FL Spectrophotometer F-4500, Tokyo, Japan) at excitation wavelengths of 340 and 380 nm and an emission wavelength of 510 nm.

### 4.4. Flow Cytometric Analysis of Surface P-selectin Expression

Washed platelets were prepared as described in the preceding subsection and aliquots of platelet suspensions (3.6 × 10^8^ cells/mL) were preincubated with the solvent control (0.1% DMSO) or morin hydrate (40 and 80 µM) and FITC-P-selectin (2 µg/mL) for 3 min; collagen (1 µg/mL) was added to trigger platelet activation. The suspensions were then assayed for fluorescein-labeled platelets by using a flow cytometer (FACScan System, Becton Dickinson, San Jose, CA, USA). Data were collected from 50,000 platelets per experimental group. All experiments were repeated at least four times to ensure reproducibility.

### 4.5. Detection of Lactate Dehydrogenase

Washed platelets (3.6 × 10^8^ cells/mL) were preincubated with the solvent control (0.1% DMSO) or morin hydrate (40, 80, 100 μM) for 20 min at 37 °C. An aliquot of the supernatant (10 µL) was deposited on a Fuji Dri-Chem slide LDH-PIII (Fuji, Tokyo, Japan) and the absorbance wavelength was read at 540 nm by using an ultraviolet–visible spectrophotometer (UV-160; Shimadzu, Japan). A maximal value of lactate dehydrogenase (LDH) was recorded in the sonicated platelets (Max).

### 4.6. Immunoblotting of Protein Phosphorylation

Washed platelets (1.2 × 10^9^ cells/mL) were preincubated with the solvent control (0.1% DMSO) or morin hydrate (40 and 80 µM) for 3 min. Subsequently, collagen (1 µg/mL) was added to trigger platelet activation. The reaction was then stopped and the platelets were immediately resuspended in 200 μL of lysis buffer. Samples containing 80 μg of protein were separated through 12% sodium dodecyl sulfate gel electrophoresis, and the proteins were electrotransferred to PVDF membranes by using a Bio-Rad semidry transfer unit (Bio-Rad, Hercules, CA, USA). The blots were then blocked through treatment with Tris-buffered saline in Tween 20 (TBST; 10 mM of Tris-base, 100 mM of NaCl, and 0.01% Tween 20) containing 5% BSA for 1 h and were probed with various primary antibodies. The membranes were incubated with HRP-conjugated anti-mouse IgG or anti-rabbit IgG (diluted 1:3000 in TBST) for 1 h. An enhanced chemiluminescence system was used to detect immunoreactive bands, whose optical density was quantified using Bio-profil Biolight (version V2000.01; Vilber Lourmat, Marne-la-Vallée, France).

### 4.7. Measurement of OH· Formation in the Platelet Suspensions or Fenton Reaction Solution Through Electron Spin Resonance Spectrometry

Electron spin resonance (ESR) spectrometry was performed using a Bruker EMX ESR spectrometer (Bruker, Billerica, MA, USA), as described previously [33]. Suspensions of washed platelets (3.6 × 10^8^ cells/mL) or the Fenton reagent (50 μM FeSO_4_ + 2 mM H_2_O_2_) were preincubated with 0.1% DMSO or morin hydrate (40 and 80 µM) for 3 min. Subsequently, collagen (1 μg/mL) was added into platelet suspensions to trigger platelet activation for 5 min. Before ESR spectrometry, 100 μM of DMPO was added to both solutions. ESR spectra were recorded using a quartz flat cell designed for aqueous solutions. The spectrometer was operated under the following conditions: power, 20 mW; frequency, 9.78 GHz; scan range, 100 G; and receiver gain, 5 × 10^4^. The modulation amplitude was 1G, the time constant was 164 ms, and scanning was performed for 42 s; each ESR spectrum obtained was the sum of four scans.

### 4.8. Measurement of Sodium Fluorescein-Induced Thrombus Formation in Mouse Mesenteric Microvessels

Male ICR mice (aged 6 weeks) were anesthetized using a mixture containing 75% air and 3% isoflurane maintained in 25% oxygen; the mice’s external jugular veins were then cannulated with a PE-10 tube for administration of dye and drugs intravenously [34]. Venules (30–40 µm) were irradiated at wavelengths of <520 nm to produce a microthrombus. Two morin hydrate doses (5 and 10 mg/kg) were administered 1 min following sodium fluorescein (15 µg/kg) administration, and the time required for the thrombus to occlude the microvessel (occlusion time) was recorded. In this experiment, the method applied to the thrombogenic animal model conformed to the Guide for the Care and Use of Laboratory Animals (8th edition, 2011), and we received an affidavit of approval for the animal use protocol from Taipei Medical University (LAC-2016-0276; 01/August/2017).

### 4.9. Measurement of Bleeding Time in Mouse Tail Vein

Bleeding time was measured through transection of the tails of the male ICR mice. In brief, after 30 min of administration of 5 or 10 mg/kg of morin hydrate intraperitoneally, the tails of the mice were cut 3 mm from the tip. The tails were then immediately placed into a tube filled with normal saline at 37 °C to measure the bleeding time, which was recorded until the bleeding completely stopped. In the animal experiments, the method applied to the animal model conformed to the Guide for the Care and Use of Laboratory Animals (8th edition, 2011), and we received an affidavit of approval for the animal use protocol from Taipei Medical University (LAC-2016-0276; 01/August/2017).

### 4.10. Statistical Analysis

The experimental results are expressed as means ± standard errors of means alongside the number of observations (*n*); *n* refers to the number of experiments; each experiment was performed using different blood donors. The unpaired Student’s *t* test was used to determine the significance of differences between control and experimental mice. Differences between groups in other experiments were assessed using an analysis of variance (ANOVA). When the ANOVA results indicated significant differences among group means, the groups were compared using the Student–Newman–Keuls method. A *p* value of <0.05 indicated statistical significance. Statistical analyses were performed using SAS (version 9.2; SAS Inc., Cary, NC, USA).

## 5. Conclusions

This paper reports that morin hydrate inhibits collagen-stimulated platelet activation by hindering the PLCγ2–PKC cascade and hydroxyl radical generation and consequently by inhibiting Akt and MAPKs activation. Together these alterations reduce the level of [Ca^2+^]i and eventually inhibit platelet aggregation. Moreover, morin hydrate does not affect platelet permeability or induce platelet cytolysis. These findings may recommend that morin hydrate establish a novel therapeutic agent for treating thromboembolic disorders.
